# Equine-assisted interventions for veterans with service-related health conditions: a systematic mapping review

**DOI:** 10.1186/s40779-019-0217-6

**Published:** 2019-08-29

**Authors:** Adam R. Kinney, Aaron M. Eakman, Rebecca Lassell, Wendy Wood

**Affiliations:** 1 Department of Occupational Therapy, Colorado State University, Fort Collins, CO 80523 USA; 2 Temple Grandin Equine Center, Department of Animal Sciences, Colorado State University, Fort Collins, CO 80523 USA

**Keywords:** Veterans, Service-related injuries, Equine-assisted interventions, Posttraumatic stress disorder, Systematic mapping review

## Abstract

**Background:**

Evidence-based treatments for service-related health conditions such as posttraumatic stress disorder (PTSD), depression, and traumatic brain injury (TBI) are not effective for all veterans. Equine-assisted interventions are emerging as an additional treatment modality, but little is known regarding the safe and effective delivery of these interventions. This study aimed to describe the following features of the body of literature concerning equine-assisted interventions among veterans: 1) veterans who have participated in equine-assisted interventions; 2) specific characteristics of equine-assisted interventions in veterans; and 3) the specific characteristics of research on equine-assisted interventions in veterans.

**Methods:**

We conducted a systematic mapping review of peer-reviewed literature reporting on equine-assisted interventions among veterans between 1980 and 2017. Searches of nine databases yielded 3336 unique records, six of which met the inclusion criteria and were reviewed. Data relevant to the study aims were extracted and analyzed.

**Results:**

Equine-assisted interventions among veterans disproportionately targeted psychosocial outcomes and yielded promising results. The detailed methods of EAI varied in the reported studies, ranging from communicating with the horse to mounted exercises. There was also great diversity in outcome measurement. The state of theoretical development regarding the mechanisms by which equine-assisted interventions benefit the veteran population is currently underdeveloped. Studies provided insufficient detail with respect to the description of the intervention, reasons for attrition, and the dose-response relationship.

**Conclusions:**

Scientific development of equine-assisted interventions targeting psychosocial outcomes among veterans is warranted to establish their efficacy. Targeted outcomes should be expanded, including outcomes more closely aligned with the nature of polytraumatic injuries. Future research must also emphasize the theoretical development of equine-assisted interventions for veterans and thoroughly describe the participants, components of the intervention, factors contributing to attrition, and optimal dose-response relationships.

## Background

In the wake of the terrorist attacks on September 11, 2001, approximately 2.77 million service members were deployed to combat zones in support of Operation Enduring Freedom, Operation Iraqi Freedom, and/or Operation New Dawn [1]. Due to psychological and physical trauma incurred during combat deployments, an influx of veterans has returned to the civilian community with *polytraumatic injuries* or complex conditions characterized by unpredictable combinations of two or more health-related diagnoses [2]. For example, those deployed to combat zones tend to experience high rates of traumatic brain injury (TBI, 10–20%), posttraumatic stress disorder (PTSD, 5.6–30.5%), and depression (5.6–16%), which tend to co-occur and pose serious threats to veterans’ cognitive and emotional functioning [3, 4]. Furthermore, the physical capacities of veterans are threatened by somatic symptoms (e.g., pain or fatigue) associated with the above conditions, as well as acquired spinal cord injuries, musculoskeletal injuries, and/or amputations [2, 3, 5].

The complex constellation of cognitive, emotional, and physical impairments experienced by veterans with polytraumatic injuries poses significant barriers to successful community reintegration [2, 6]. Veterans with TBI, PTSD, and/or depression experience difficulties with establishing supportive and close connections with others [7, 8]. Furthermore, veterans with polytraumatic injuries experience limited engagement in community-based activities, thereby compromising their successful transition to civilian life [9, 10]. Veterans report that cognitive and emotional impairments associated with these conditions foster a tendency to avoid community-based activities in the community given the presence of others [11, 12] and that physical symptoms (e.g., pain or fatigue) disrupt veterans’ participation in typical patterns of activity [11, 13].

The development of treatments for service-related health conditions such as PTSD, depression, and TBI has notably progressed. For example, symptoms of PTSD have been shown to improve in response to both exposure-based therapy [14] and cognitive processing therapy [15]. These treatments are not effective for all veterans, however. Evidence suggests, for instance, that a significant proportion of participants continue to meet diagnostic criteria for PTSD despite receiving established treatments such as exposure-based therapy [15, 16]. Furthermore, these treatments tend to target service-related health conditions in isolation, and as such, there is a significant need for additional approaches that transcend specific diagnoses and are tailored to address the multifaceted concerns of veterans with polytraumatic injuries [17]. The formidable challenges associated with polytraumatic injuries thus warrant the application of interventions that are similarly complex, such as equine-assisted interventions.

### Equine-assisted interventions

The term *animal-assisted interventions* (AAIs) is a commonly used umbrella term that encompasses a plethora of ways in which different species of animals are beneficial to people [18]. *Equine-assisted interventions,* another umbrella term, comprise a growing subset of AAIs and encompass both equine-assisted activities (EAAs) and equine-assisted therapies (EATs). Broadly speaking, EAAs involve horses, clients, participants, volunteers and instructors affiliated with an equine center in mounted activities on a horse, as well as unmounted activities such as grooming, tacking or caring for a horse [19]. Whereas EATs also integrate activities involving horses, credentialed health professionals design, deliver, or direct these goal-directed interventions in accord with their professions’ respective scopes and standards of practice [19].

Between 2009 and 2016, the number of equine centers accredited by the Professional Association of Therapeutic Horsemanship International (PATH Intl) providing services to veterans grew from 89 to 335 centers, which is more than a three-fold increase [20]. Based on research of equine-assisted interventions for other populations [21, 22], the services that these equine centers offered to veterans were presumably complex in nature; that is, they comprised complex interventions. *Complex interventions* include but are not limited to the following features: 1) the presence of several interacting components, 2) the ability to easily tailor the intervention to meet appropriate objectives, and 3) the capability of addressing highly variable outcomes [23]. Due to these features, complex interventions may be uniquely situated to address the multifaceted and heterogeneous nature of challenges associated with polytraumatic injuries among veterans [2]. Indeed, equine-assisted interventions for civilian populations with conditions often experienced by veterans have shown promising results, suggesting that they could yield similarly positive results among veterans. For example, therapy involving horses has been found to improve 1) function for those with traumatic brain injury [24], 2) psychosocial outcomes for those with depression [25], 3) psychological well-being among those with spinal cord injuries [26] and 4) symptoms of PTSD [27].

Considered altogether, the proliferation of EAAs and EATs for veterans with service-related health conditions begs for deliberate efforts aimed at advancing their theoretical and empirical bases. To the best of our knowledge, however, relevant peer-reviewed literature has not been systematically collected, categorized, described, and synthesized. Prior to evaluating complex interventions such as equine-assisted interventions, it is critical that a systematic review of the evidence germane to the intervention and its intended population is conducted [23]. In pursuit of this outcome, the present study has three specific aims:

1. To describe the characteristics of veterans (i.e., health conditions, gender, and age) who have participated in equine-assisted interventions.
2. To describe the specific features of equine-assisted interventions which have been applied to the veteran population, including the (a) prevalence of specific types of EAA and EAT, (b) intervention components, (c) providers, and (d) length, frequency, and/or duration of intervention sessions.
3. To describe the specific characteristics of research on equine-assisted interventions in the veteran population, including the (a) study designs, (b) reported outcomes and benefits, and (c) theoretical explanations of benefits.

## Methods

Representing one of the 14 types of reviews in the family of systematic review research, systematic mapping reviews are a method of choice when research in a focused area of inquiry is early in scientific development but has yet to be systematically gathered, described, and categorized, in short, mapped [28, 29]. Researchers conducting systematic mapping reviews employ a broad scope, drawing from research reports with varying degrees of rigor to construct detailed classifications of a particular body of literature. While the quality of evidence is not assessed in a systematic mapping review, the employment of such a broad scope makes this method of review ideal when summarizing areas of inquiry in the early stages of development, including inquiry concerning the development and evaluation of complex interventions [23]. Systematic mapping reviews require the application of three filters to the existing literature: 1) gathering the literature, 2) selecting papers for inclusion, and 3) extracting information from selected papers that correspond to specific research aims.

### Search method

The search procedure for the present study is consistent with the search procedure reported in previously developed papers [21, 30]. Using approximately 45 search criteria and beginning in 2015, a library scientist developed and executed four comprehensive searches in the following databases to assist in multiple projects regarding equine-assisted activities or therapies: CAB Abstracts (EBSCO), CINAHL (EBSCO), PsycINFO (EBSCO), PubMed (NCBI), Social Sciences Abstracts (EBSCO), Social Services Abstracts (ProQuest), Social Work Abstracts (EBSCO), SPORTDiscus (EBSCO), and Web of Science (Thomson Reuters). The final search was conducted in spring 2018 to retrieve papers published through 2017. The results of all searches were integrated into one EndNote library for screening, which facilitates efficient organization and management of references [31]. The search procedure yielded 3336 unique records.

### Application of inclusion and exclusion criteria

Three reviewers reviewed papers for inclusion and exclusion criteria in two phases. In *phase one*, we applied the following inclusion criteria: papers were 1) primarily focused on one or more equine-assisted activity or therapy, 2) peer-reviewed, 3) a primary source, 4) written in English, and 5) published between 1980 and 2017. The reviewers blindly analyzed 20% of all sources initially retrieved, achieving 95% agreement on their respective inclusion or exclusion decisions. Reviewers subsequently applied inclusion and exclusion criteria to the 3336 unique records, plus reviewed the reference lists of included papers for other relevant articles. This yielded an additional 180 unique records. Of the 3516 unique records identified through the search procedure and manual searching of reference lists, 506 remained after the first phase of review.

**Fig. 1:**
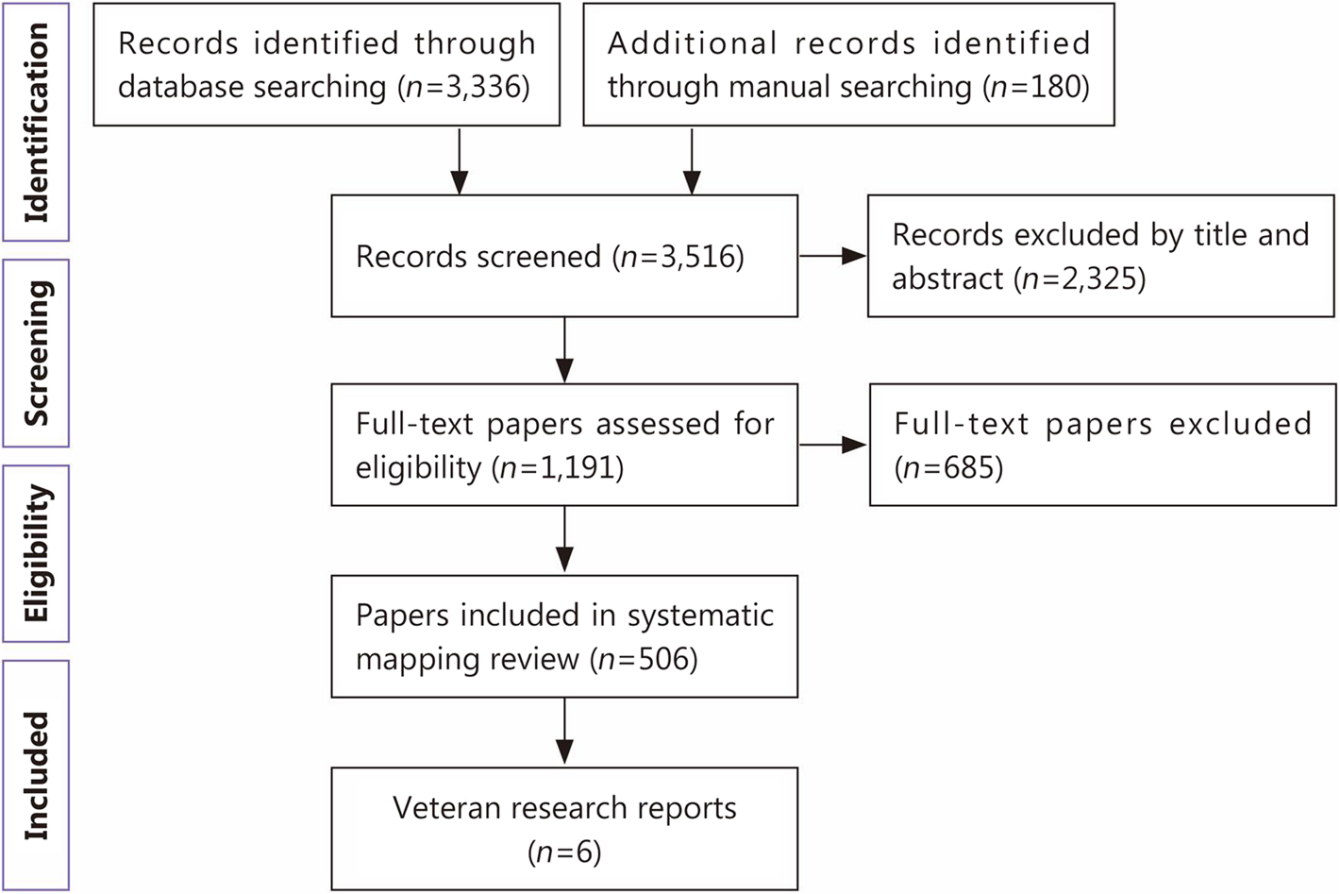
Flow diagram of systematic mapping review and included studies

In *phase two*, we applied additional exclusion criteria to further reduce the 506 papers. Specifically, papers were excluded if 1) 20% or more of the participants were not military veterans or 2) the paper provided a synopsis of an EAAT-related study published elsewhere. Application of the above criteria reduced the 506 records to eight records pertaining to EAAT for veterans. These eight records were composed of six original research reports and two conceptual or descriptive papers. We extracted data from the six original research reports to address the specific aims of this systematic mapping review (Fig. 1).

### Extraction of data

Consistent with the established protocol for systematic mapping reviews [32], we constructed a data extraction tool (DET) to facilitate the extraction of information relevant to each study aim.

#### Aim 1

We applied the DET to extract information related to: 1) age, 2) proportion of males, 3) health conditions of participants, and 4) assessment tools used to describe participants.

#### Aim 2

We employed the DET to extract information related to: 1) the specific type of EAA or EAT studied, 2) participant goals, 3) specific components of the intervention, 4) direct providers of the intervention, and 5) lengths and frequencies of individual sessions and overall duration of the intervention.

#### Aim 3

We applied the DET to extract information related to: 1) theoretical propositions proffered to explain the benefits of interventions, 2) research aims, 3) research approach (e.g., quantitative, qualitative, mixed or multi-methods), 4) specific research design (e.g., one group pre-post design), 5) method of measurement (e.g., interview, standardized assessment), and 6) reported outcomes.

Also related to Aim 3, the DET provided guidelines for sorting reported outcomes into broad categories. Four of these broad categories classified the focus of targeted outcomes: 1) body functions, 2) activity and participation, 3) symptoms of mental disorders, and 4) other outcomes. The International Classification of Functioning, Disability, and Health (ICF) defines *body functions* (ICF-BF) as physiological functions of the body (e.g., cognitive functions such as attention)*, activity* as the performance of a particular task or action, and *participation* as “involvement in a life situation” (p. 10) [33]. The DET combined activity and participation into one level of functioning, ICF-A/P, because distinctions between activity and participation are difficult to establish [33]. The category *symptoms of mental disorders* was derived from the Diagnostic and Statistical Manual of Mental Disorders, Fifth Edition (DSM-V) [34]. The category *other outcomes* included outcomes that did not align with the above three broad categories (e.g., learning about oneself). Three other broad categories were used to sort outcomes based on the nature of the reported findings: 1) statistically significant findings or findings that reflected hypothesized positive outcomes and included supporting statistical evidence, 2) other positive findings or findings reported as clinically important but did not include statistical evidence to support their existence, and 3) negative findings or findings that were not reported as statistically significant or clinically important.

A research consultant entered the finalized DET into a Microsoft Access database. Using this database, the last author directed a training process that produced a minimum of 90% agreement on use of the DET across six reviewers. Inter-rater reliability was also supported by the establishment of Kappa coefficients, which ranged between 0.65 and 0.74. Inter-rater reliability checks were conducted consistently to protect against intra- and inter-rater drift. We also used the query tool in Microsoft Access to analyze specific components of the DET that corresponded with specific study aims. Subsequently, we exported those results to Microsoft Excel and employed the pivot table function to establish descriptive statistics (i.e., frequency counts and proportions) that pertained to each research aim.

## Results

We used five tables to map the key findings of this systematic mapping review. Table 1 details the aims of the six included studies and the characteristics of veteran participants in each study (Aim 1). Table 2 identifies different types of EAAs and EATs that were offered to veterans and presents characteristics of the studied interventions, including identified dosages, providers, and components of the intervention package (Aim 2). Table 3 elucidates each intervention component (Aim 2). Table 4 presents the study designs, outcome measures, and classifications of reported findings pertaining both to their focus and the levels of significance (Aim 3). Table 5 summarizes the theorized benefits of each study (Aim 3). We next elaborate on these findings in more detail.

**Table 1:** General descriptions of the six filtered studies that primarily focused on equine-assisted interventions

1st author (year) Country	Study aims	Diagnoses and descriptors	Number of participants	Number of male participants (%)	Age (year)
Lanning (2013) United States	To address the need for research in EAA by assessing the changes in quality of life indicators and depression symptoms of veterans participating in a PATH International Equine Service for Heroes TR program	PTSD, sexual abuse/trauma, veteran, physical disabilities	13^a^	10 (76.9)	29–52
Nevins (2013) United States	To investigate the possible psychological and psychosocial benefits of teaching a post deployed veteran the Saratoga WarHorse Connection method	Veteran, PTSD, depression, student	1	1 (100.0)	52
Duncan (2014) Canada	To assess the benefits of participating in a PTSD-tailored EAL program by measuring the acquisition of knowledge in interpersonal skills, self-mediation, and perceived relief from PTSD symptoms during the EAL session using the HOLSTER and BELT scales	Veterans, PTSD, OSI	58	56 (96.6)	–
Aldridge (2016) United States	To compare traditional physical therapy to hippotherapy combined with traditional physical therapy on the motor performance of a 34-year-old male military veteran with low back pain.	Veteran, PTSD, low back and neck pain	1	1 (100.0)	34
Ferruolo (2016) United States	To analyze deidentified self-report evaluation data from participants.	Veterans	8	8 (100.0)	–
Lanning (2017) United States	To examine the effects of THR on PTSD symptoms, quality of life, and functioning within the ICF framework.	Veterans, PTSD	51	33 (64.7)	22–57

^a^Author provided characteristics of the 13 participants who began the intervention, not the seven who completed the entire intervention; *BELT* Benefitting from Experiential Learning Together, *EAA* Equine-assisted activities, *EAL* Equine-assisted learning, *HOLSTER* Horses Relieving Operational Stress through Experiential Relationships, *ICF* International Classification System, *PATH* Professional Association of Therapeutic Horsemanship, *PTSD* Posttraumatic stress disorder, *THR* Therapeutic horseback riding, *OSI* Operational stress injury, *TBI* Traumatic brain injury. -. Not available

**Table 2:** A detailed features of equine-assisted interventions in the six filtered studies

Items	Lanning (2013)	Nevins (2013)	Duncan (2014)	Aldridge (2016)	Ferruolo (2016)	Lanning (2017)
Type of EAA or EAT						
EAA	THR	NOS	EAL	–	–	THR
EAT	–	–	–	HPOT	EFMH	–
Provider	–	CT-NOS	–	PT	SW EFMHE	TRI
Sessions						
Duration	24 weeks	3 days	–	15 weeks	1 week	8 weeks
Number of treatment times	24	3	–	15	2	8
Length	1–2 h	4 h	–	1 h	7 h	1.5 h
Body language and communication	✓	✓	✓	–	✓	✓
Ride the horse	✓	–	–	✓	✓	✓
Groundwork	✓	✓	–	–	✓	✓
Groom the horse	–	–	–	✓	✓	✓
Get to know the horse	–	✓	–	–	✓	✓
Matching horse and participant	✓	–	–	–	–	✓
Care for the horse	–	–	–	✓	–	✓
Social activities	✓	–	–	–	–	✓
Mounted exercises	–	–	–	✓	✓	–
Tack the horse	–	–	–	✓	–	–
Safety behaviors	–	✓	–	–	–	–
Connections to daily life	–	–	–	–	✓	–
Family participation	–	–	✓	–	–	–
Integration of therapeutic practices	–	–	–	–	✓	–

*EAA* Equine-assisted activities, *EAL* Equine-assisted learning, *EAT* Equine-assisted therapy, *EFMH* Equine facilitated mental health, *HPOT* Hippotherapy, *NOS* Not otherwise specified, *THR* Therapeutic horseback riding, *CT-NOS* Certified trainer not otherwise specified, *EFMHE* Equine facilitated mental health expert, *PT* Physical therapist, *SW* Social worker, *TRI* Therapeutic riding instructor; ✓ Present; −. Not present

**Table 3:** Detailed descriptions of various equine-assisted interventions involved

Component	Description
Body language and communication	Verbal and nonverbal language and body language towards horses or humans
Ride the horse	Mounted activities including walking, trotting, cantering, steering, riding around an obstacle course, etc.
Groundwork	Unmounted activities that involve leading the horse with or without the halter, work in the round pen, leading the horse around an obstacle course, ground exercises such as stopping, turning, backing up, etc.
Groom the horse	Grooming, bathing, picking hooves
Get to know the horse	Observing the horse in the pasture or stall, intentional time spent becoming acquainted with the horse
Matching horse and participant	Intentional matching, either participant chooses horse or the instructor/therapist matches horse to the participant
Care for the horse	Feeding, cleaning stalls, turning a horse out or in from pasture, barn work related to horse care or article specifically referencing caring for a horse
Social activities	Intentional activities to promote socialization such as sharing a meal with other veterans or pairing a veteran volunteer with a participant
Mounted exercises	Participant completes stretching and strengthening exercises while riding the horse or rides in different positions (sitting backwards, sideways, or prone)
Tack the horse	Activities involving putting tack (bridle, saddle pad, saddle) on or untacking
Safety behaviors	Activities focused on safety around the horses
Connections to daily life	Relating concepts learned from activities with the horse to the participant’s daily life, this could be through conversation or metaphor
Family participation	Spouses or family partners participated during the session
Integration of therapeutic practices	Implementing other therapeutic methods into EAAT practice such as cognitive behavioral therapy, mindfulness-based stress reduction, motivational interviewing, or reality testing

**Table 4:** Study designs and outcomes of equine-assisted interventions in the six filtered studies

1st author (year)	Study design	Assessment describing population	Outcome measures	Outcome classifications and levels of significance	Reported outcomes
Lanning (2013)	Mixed methods	–	BDI-II	◇ DSM-V: depression	Decreased symptoms
			SF-36v2	◇ Other	Quality of life: Physical component (i.e., physical functioning, role physical, general health); mental component (i.e., vitality, social functioning, role emotional, mental health)
				—Other	Quality of life: Bodily pain
			Semi-structured Interview	◇ ICF-AP: Interpersonal	Increased sociability (i.e., forming new relationships), increased trust in others, more open and accepting of others
				◇ ICF-AP: Community Life	Decreased isolation
				◇ ICF-BF: Temperament	Increased confidence
				◇ Other	Stronger
Nevins (2013)	Case study	CAPS	BDI-II	◇ DSM-V: Depression	Decreased symptoms
			MSSS	◇ Other	Increased perceived social support
			PCL-C	◇ DSM-V:PTSD	Decreased symptoms
			QOLI	—Other	Fluctuating changes in dissatisfaction, happiness, and satisfaction
			RSES	◇ Other	Resiliency: increased from baseline to 4- and 12-week posttests
Duncan (2014)	1 group pre-post	–	BELT	◇ DSM-V:PTSD	Decreased symptoms as reported by partner
				◇ ICF-BF: Temperament	Increased coping self-efficacy as reported by partner
				◇ Other	Partners’ feelings towards EAL program
			HOLSTER	◇ DSM-V:PTSD	Decreased symptoms as reported by participant
				◇ ICF-BF: Temperament	Increased coping self-efficacy as reported by participant
Aldridge (2016)	Single-subject design	–	NDI	◇ ICF-BF: Pain	Clinically significant reduction in neck pain
			OLBPQ	◇ ICF-BF: Pain	Clinically significant reduction in low back pain
			SDS	♦ Other	Reduced disability
Ferruolo (2016)	–	–	Open-ended questions	◇ ICF-AP: Interpersonal	Improved trust, improved respect for others
				◇ Other	Learned about self, spiritual connection
			Questionnaire	◇ DSM-V: Depression	Self-reported decreased symptoms
				◇ DSM-V: Anxiety	Self-reported decreased symptoms
				—Other	No differences in outcomes between those attending 1 day versus 2 days of program
Lanning (2017)	Mixed-methods, repeated measures	PCL-M	PCL-M	♦ DSM-V: PTSD	Decreased symptoms
			SF-36v2	♦ Other	Quality of life: mental component (i.e., vitality, social functioning, role emotional, mental health)
				—Other	Quality of life: physical component (i.e., physical functioning, role physical, general health)
			WHODAS	◇ ICF-AP: Interpersonal	Increased understanding and communication, getting along with people, participating in society
				◇ ICF-AP: Self-care	Increased self-care
				◇ Other	Reduced disability
			Semi-structured Interview	◇ DSM-V: Anxiety	Reduced symptoms
				◇ ICF-BF: Temperament	Improved confidence, increased hope
				◇ ICF-BF: Attention	Attending to the present time
				◇ ICF-AP: Interpersonal	Acceptance of self and others, gratitude, increased trust, improved patience, improved ability to set boundaries
				◇ ICF-AP: Recreation	Working with horses
				◇ ICF-AP: Non-remunerative employment	Volunteering for program

*CAPS*. Clinician Administered PTSD Scale, *CSES* Coping Self Efficacy Scale, *DERS* Difficulties in Emotion Regulation Scale, *DQ* Demographic Questionnaire, *PCL-M* PTSD Checklist Military Version, *BDI-II* Beck Depression Inventory II, *BELT* Benefitting from Experiential Learning Together, *HOLSTER* Horses Relieving Operational Stress through Experiential Relationships, *MSSS* Modified Social Support Survey, *NDI* Neck Disability Index, *OLBPQ* Oswestry Low Back Pain Questionnaire, *PCL-C* PTSD Checklist Civilian Version, *QOLI* Quality of Life Inventory, *RSES* Response to Stressful Experiences Scale, *SDS* Sheehan Disability Scale, *SF-36v2* SF-36v2 Quality of Life Assessment, *WHODAS* World Health Organization Disability Assessment Schedule, *DSM-V* Diagnostic & statistical manual of mental disorders-V, *ICF-AP* International Classification of Functioning, Disability, and Health (ICF)-Activity/Participation, *ICF-BF* ICF-Body Functions. ♦. Statistically significant finding; ◇. Other positive finding; —. Negative finding

**Table 5:** Theoretical propositions to explain the benefits of equine-assisted interventions in the six filtered studies

Items	Lanning (2013)	Nevins (2013)	Duncan (2014)	Aldridge (2016)	Ferruolo (2016)	Lanning (2017)
Type of EAA or EAT	THR	NOS	EAL	–	–	THR
EAA						
EAT	–	–	–	HPOT	EFMH	–
Horse-human interaction/bond	✓	✓	–	–	–	✓
Socialization with others/group reflection	✓	✓	–	–	✓	–
Physical features of the equine environment	–	✓	–	–	–	✓
Horse as a mirror or metaphor	–	–	–	–	✓	✓
Being in the moment/mindfulness	–	–	✓	–	✓	–
Safe and nonjudgmental environment	–	–	✓	–	–	✓
Experience of control/autonomy	✓	✓	–	–	–	–
Opportunity to practice communication	–	–	✓	–	–	–
Movement of the horse	–	–	–	✓	–	–
Fosters motivation	–	–	–	✓	–	–
Cognitive Behavioral Therapy	–	–	–	–	✓	–
Calming effect of the horse	✓	–	–	–	–	–
Motivational interviewing	–	–	–	–	✓	–
Nontraditional therapy setting	–	–	–	–	–	✓

*THR* Therapeutic horseback riding, *NOS* Not otherwise specified, *EAL* Equine-assisted learning, *HPOT* Hippotherapy, *EFMH* Equine facilitated mental health; ✓. Present; −. Not present

### Aim 1: characteristics of veteran participants

Five of the six included studies reported participants’ health conditions (Table 1). PTSD was reported in all of these five studies, thereby representing the most frequently reported condition. Across the five studies that reported health conditions, PTSD was followed by physical health conditions (two studies), depression (one study), sexual abuse/trauma (one study), and operational stress injury (one study). Four of these studies included participants with multiple health conditions (e.g., PTSD and physical health conditions). Furthermore, two studies were case studies of a veteran experiencing multiple health conditions *concurrently*. Of the five studies that reported health outcomes, two employed standardized instruments to describe the sample in terms of PTSD symptomatology.

All six studies reported information on participant gender; the majority of veteran participants were male, with proportions ranging from 64.7 to 100%. Four of the six studies reported the age of participants. Veterans across a large portion of the lifespan participated in the seven studies (age range: 22–73 years).

### Aim 2: specific characteristics of equine-assisted activities or therapies

#### Types of equine-assisted activities or therapies

Four of the six included studies investigated interventions classified as EAAs (Table 2). Across these four studies, two investigated therapeutic horseback riding (THR). The average duration of THR was 16 weeks, although considerable dosing variations across the two studies were evident. Only one study of THR explicitly identified a provider [35]. Additionally, across the four studies of EAAs, one study investigated equine-assisted learning (EAL) [36]. This study was conducted in Canada and was the only study conducted outside the United States. While EAL was identified as consisting of three daily four-hour sessions, the duration, number, or length of the EAL was not provided, nor was the provider identified. An EAA that was not otherwise specified (NOS) was investigated in one of the four studies; this study sought to understand the benefits of the Saratoga WarHorse Connection method and identified the provider as a certified trainer [37].

Two of the six studies included investigated the EATs of hippotherapy (HPOT); [38] and Equine-facilitated Mental Health (EFMH) [39]. Both of these studies were conducted in the United States. As illustrated in Table 2, the identified providers and dosages of these two EATs differed substantively.

#### Components of equine-assisted interventions

All six studies described components of the intervention (Tables 2 and 3). The number of components described within these studies ranged from two [36] to eight [35, 39], with an average of approximately five components (*M* = 5.33, *SD* = 2.34). Generally, the number of components did not differ substantially when comparing the four studies of EAAs (*M* = 4.75, *SD* = 2.50, min – max: 2–8) to the two studies of EATs (*M* = 6.5, *SD* = 2.12, min – max: 5–8).

The most commonly employed component across the six studies was practicing body language and communication (five of the six studies), followed by riding the horse and groundwork (four of the six studies). While all studies described these and other components, descriptions of the components were generally void of detail. For example, a recent study of THR devoted approximately one paragraph simply to listing components such as riding the horse, grooming the horse, and matching the horse with the participant [35]. Furthermore, the modes of intervention delivery were described in only three studies, with two studies [37, 38] specifying an individual session and one study [39] specifying a group session.

While the small number of included studies makes it difficult to discern definitive patterns of differences between EAAs and EATs with respect to intervention components, some differences warrant brief description. Whereas all four studies of EAAs identified practicing body language and communication skills as an intervention component (four studies), only one of the two studies of EAT identified this component. Furthermore, three of the four studies of EAAs emphasized groundwork, while only one of the two studies of EATs included this component. Similarly, two of the four studies of EAAs included matching the horse with participants and social activities compared with none of the two EAT studies. Conversely, while both studies of EATs identified grooming the horse as an intervention component, only one of the four studies of EAAs did so. Both studies of EATs identified riding the horse as a salient aspect of the intervention compared to two of four studies of EAAs. Finally, the component of mounted exercise was employed in both studies of EATs but was absent among all four studies of EAAs.

### Aim 3: characteristics of research on equine-assisted interventions for veterans

#### Study designs

Five of the six included studies explicitly labeled their study design (Table 4). Of these five studies, all studies used designs consistent with early phase research (i.e., quasi-experimental designs and/or small sample sizes; [40]). Specifically, two of the five studies employed a mixed methods approach integrating a one-group pre-post design, two studies used a single-subject case design, and one study used a one-group pre-post design. While these studies were quasi-experimental, two [35, 36] included relatively robust sample sizes (58 and 51 participants), while the remaining had relatively small sample sizes (one to 13 participants). Whereas all six studies analyzed outcomes quantitatively, two studies used a mixed-methods approach that included qualitative analyses of veterans’ experiences of the interventions [35, 41]. While one study [39] did not explicitly label its design, our review found that the authors used qualitative data (i.e., responses to open-ended questions) in concert with a descriptive questionnaire (e.g., dichotomous items indicating whether the program eased participant anxiety) to explore the participants’ experience of the intervention.

#### Classifications of reported outcomes

Four of the six included studies reported specific outcomes that we classified at the ICF-BF level of functioning, most of which pertained to the temperament and personality domain of body functions. Specifically, two studies reported increased confidence, one study reported increased hope, and one study reported outcomes related to coping self-efficacy (from the perspective of both the participant and the participant’s partner). One study assessed the attention domain of body functions concerning attending to the present time [35]. The only study that assessed an outcome at this level of functioning pertaining to physical or sensory domains was the study by Aldridge and colleagues [38], which reported outcomes with respect to both neck and back pain. We classified all outcomes at the ICF-BF level of functioning as promising.

Three of the six included studies reported outcomes that we classified at the ICF-AP level of functioning. These outcomes emphasized psychosocial-related phenomena, particularly interpersonal functioning. Outcomes related to interpersonal functioning were heterogeneous and included (but were not limited to) outcomes such as trust in others (three studies), skills related to effective communication (e.g., patience, respect for/getting along with others; three studies), acceptance of oneself and/or others (two studies), and the ability to form new relationships (one study) [35, 39, 41]. Other reported outcomes consistent with the ICF-AP level of functioning concerned community life (i.e., isolation; one study), self-care (one study), recreation (i.e., continued working with horses; one study), and nonremunerative employment (i.e., continued volunteering for the program; one study). Overall, we classified 100% of all outcomes consistent with the ICF-AP level of functioning as promising findings.

All six studies included at least one outcome categorized as other, and these outcomes were highly variable. Outcomes classified as “other” included but were not limited to quality of life (three studies), disability (two studies), social support (one study), resiliency, learning about oneself, and feelings regarding the intervention itself. The 13 outcomes classified as other were also generally positive, with two outcomes reflecting statistically significant findings, seven outcomes reported to be otherwise promising findings, and the remaining four outcomes classified as negative findings.

Finally, five of the six studies reported outcomes that we classified as relevant to the DSM-V. Of these five studies, PTSD and depressive symptoms were most commonly addressed (three studies each). One study [36] assessed PTSD symptomatology from the perspectives of the participant *and* the participant’s partner. Symptoms of anxiety were the only other reported outcome that was classified as relevant to the DSM-V and was addressed in two of the five studies. Studies that assessed outcomes consistent with the DSM-V revealed positive findings, with one of the nine findings reflecting a statistically significant reduction in PTSD symptoms [35]. The remaining eight outcomes consistent with the DSM-V were considered to be otherwise promising findings.

#### Theoretical explanation of benefits

Fourteen fairly heterogeneous explanations of the benefits of interventions were found across the six studies (Table 5). Individual studies also offered multiple explanations of the benefits of interventions (*M* = 3.83, *SD* = 1.17, min – max: 2–5). The most commonly reported explanations for positive outcomes pertained to veterans’ interactions or bonds with horses and socializing with other humans (three studies). All three studies that invoked the theoretical mechanism of therapeutic bonds between humans and horses were studies of EAAs, two of which studied THR. Furthermore, five of the six studies invoked features of the physical or social environment to explain the positive benefits, such as the aforementioned therapeutic mechanism of socializing with other humans. Two studies also discussed the safe and nonjudgmental nature of the equine context. Furthermore, two studies posited that physical features of the equine environment (e.g., barn, stable, or natural environment) facilitated positive effects.

Although there was relatively little consensus concerning other proposed therapeutic mechanisms, they warrant description. Two of the six studies proposed that using the horse as a mirror or metaphor, being in the moment or being mindful, and experiencing a sense of autonomy explained the positive benefits of the studied interventions. One study employed established therapeutic approaches (i.e., cognitive behavioral therapy and motivational interviewing) to explain positive outcomes. Other explanations invoked in only one study included harnessing the horse’s movement to stimulate sensorimotor functions, enhancing motivation, affording opportunities to practice communication skills, the calming effect of the horse, and the nontraditional nature of the equine-assisted therapy setting.

## Discussion

Using the method of a systematic mapping review, we found promising evidence that some EAAs and EATs may benefit veterans with service-related injuries, in particular those that targeted psychosocial outcomes. At the same time, the body of literature that provided this evidence is sparse and reflects very early scientific development. We next elaborate on the nature of promising findings, as well as gaps in knowledge and needed future research steps.

Established treatments that target psychosocial outcomes such as exposure-based therapy are not effective for up to 50% of veterans [15, 16]. The evidence gleaned through this systematic mapping review indicates that equine-assisted interventions warrant continued scientific investment to establish their efficacy as additional treatment approaches for veterans with psychosocial difficulties. For example, an included study of THR observed statistically significant reductions in veterans’ experience of PTSD symptoms and a corresponding improvement in general psychosocial functioning (e.g., improved social functioning and reduced barriers to role fulfillment) [35]. Other promising findings indicate a positive impact of the Saratoga WarHorse project, EAL, and EFMH on depressive symptoms [37, 39], coping ability [36], and anxiety [35, 39].

While all studies employed some form of quantitative methods, three studies [35, 39, 41] integrated qualitative data, thereby shedding light on veterans’ subjective experience of equine-assisted interventions. Qualitative findings substantiated the aforementioned psychosocial benefits by documenting decreased anxiety [35] and improved social functioning (e.g., improved ability to form new relationships; [41]). In addition, qualitative methods yielded insight into an expanded view of outcomes that may be targeted in future inquiry. In particular, all three of these studies offered a more refined view of the benefits of equine-assisted interventions on social functioning. Veterans reported that the interventions resulted in increased trust, patience, gratitude, and respect for others, as well as an improved ability to set boundaries during social interactions. This refined knowledge of how equine-assisted interventions may benefit social functioning reflects an important contribution to the existing literature by encouraging a more precise assessment of outcomes, thereby fostering ongoing theoretical and empirical development.

Qualitative findings also yielded interesting results with respect to self-referential outcomes and suggest that equine-assisted interventions may encourage veterans to endorse a more positive appraisal of themselves and their relationship with the world. For example, veterans in two studies [35, 41] reported that the intervention improved their overall confidence. In addition, veterans reported learning about themselves [39] and an increased acceptance of themselves [35]. Importantly, outcomes that reflect a more positive appraisal of themselves (e.g., self-efficacy and self-esteem) have been identified as a fruitful target for rehabilitative efforts [42] and are considered critical for psychological wellbeing [43, 44]. Increased attention to self-referential outcomes such as self-efficacy may be particularly important to veterans, many of whom negatively appraise their abilities as significant barriers to community reintegration [45]. Future studies of equine-assisted interventions for the veteran population should therefore emphasize self-referential outcomes within theoretical and empirical development efforts.

Finally, qualitative findings indicated that equine-assisted interventions may produce improved engagement in the civilian community, which veterans indicate is a common challenge upon detachment from the military [9, 11]. For example, veterans in the included studies reported a decreased sense of isolation [41] and increased engagement in recreational activities and volunteering [35]. These qualitative findings, along with the aforementioned findings with respect to improved role fulfillment [35], suggest that equine-assisted interventions may be capable of supporting veterans’ community reintegration, a rehabilitative outcome of utmost importance for the veteran population [46, 47]. However, the majority of the included studies did not consider the impact of the intervention on *individualized* patterns of engagement in the civilian community or the extent to which those patterns of engagement aligned with veterans’ values and interests. For example, for veterans with children, a relevant and valued outcome may be shared engagement in activities with their children. Conversely, this same outcome may not be relevant for veterans *without* families. Future studies should target and assess outcomes related to the individualized and subjective experience of engagement in the community because engagement in activities that align with one’s personal values and interests is considered a critical ingredient for psychological wellbeing [48, 49].

### Next research steps

Future studies may benefit from targeting a more expansive range of outcomes including but not limited to outcomes consistent with the complex and unpredictable nature of polytraumatic injuries. Future studies must also address gaps with respect to theoretical development, descriptions of participants and the intervention, factors influencing attrition, and the dose-response relationship.

The reviewed studies reflected a disproportionate emphasis on the influence of equine-assisted interventions on psychosocial outcomes and generally overlooked the multifaceted concerns of veterans with polytraumatic injuries that transcend both psychosocial *and* physical domains (e.g., pain; [5, 50]). Clearly, psychosocial functioning is a critical target for intervention, but the physical challenges faced by veterans similarly disrupt their successful transition to civilian life [11, 13]. For example, the rates of pain among returning post-9/11 veterans are alarming, ranging from 47 to 89% [51, 52]. Furthermore, pain tends to co-occur with psychiatric conditions such as PTSD, with one study documenting that 66% of their sample with PTSD reported comorbid chronic pain [53]. Unfortunately, despite evidence suggesting that equine-assisted interventions can positively influence physical domains such as pain [54–56], our review found only one study that investigated similar outcomes in the veteran population. This single-subject study [38] documented positive results, including reductions in pain and corresponding reductions in disability. While this study yielded promising results, there is a persistent need for the development of equine-assisted interventions among the veteran population that fully unleash their potential as complex interventions [21–23]. Specifically, future efforts should develop an understanding of how to precisely tailor equine-assisted interventions to address the complex and individualized patterns of psychosocial and physical impairments experienced by veterans with polytraumatic injuries [2].

While the existing evidence offers promise for the efficacy of equine-assisted interventions for the veteran population, it is imperative that empirical support for such interventions continues to develop. The paucity of studies investigating the impact of equine-assisted interventions for veterans does not correspond to the rapid proliferation of such interventions available to veterans [20]. The state of the evidence is most consistent with early phases of scientific development, consisting of quasi-experimental and case study designs [23, 40]. Ongoing establishment of such evidence is critical, especially given that there were over 300 programs delivering equine-assisted interventions for veterans in 2016 [20]. Notably, while they did not meet our inclusion criteria due to their publication after 2017, three recent studies [57–59] employed controlled trial designs, which are methods consistent with more mature phases of scientific development. These studies bolstered reviewed findings by observing statistically significant reductions in PTSD symptoms. However, our review revealed that significant gaps in knowledge remain and must be addressed to facilitate the identification of interventions most worthy of widespread implementation among veterans.

A foundational component of developing and evaluating an intervention is to propose and test the underlying theory concerning the mechanism by which an intervention influences targeted outcomes [23, 40]. Theoretical development is a necessary step that informs translation of research findings into practice by shedding light on *how* the intervention produces change and for *whom*, thereby guiding efforts to refine the intervention to ensure its safe and effective delivery [60]. Unfortunately, the state of theoretical development regarding the mechanisms by which equine-assisted interventions benefit the veteran population is currently insufficient. The present review summarized authors’ proposals of 14 distinct mechanisms by which equine-assisted interventions benefit veterans, highlighting the lack of consensus across the body of literature. There is a need for deliberate efforts to foster a more parsimonious understanding of the proposed benefits of equine-assisted interventions among the veteran population, thereby enabling more focused inquiry into the mechanisms by which observed outcomes are achieved and more precisely guiding efforts that maximize the interventions’ efficacy.

While all of the included studies offered theorized explanations for the benefits of equine-assisted interventions for veterans, few integrated their proposed mechanisms into the study design in a way that offered empirical support for their propositions. While it was not their stated objective, only three studies [35, 38, 41] used qualitative data to access veterans’ experience of the intervention, thereby offering evidence capable of advancing theoretical development [61]. Future inquiry should involve 1) proposing a particular therapeutic mechanism (e.g., interacting with the horse fulfills emotional needs), 2) testing the intervention’s ability to elicit those benefits (e.g., assessing emotional needs fulfillment during intervention), and 3) attempting to link those benefits to targeted outcomes. Engaging in such a process is a critical component of assessing intervention effects and would help advance the iterative process of theoretical development by discarding proposed mechanisms that fail to garner empirical support [62, 63].

Existing evidence offers glimpses of potentially fruitful targets for theoretical development. Five out of six studies identified a feature of the social and/or physical environment as a potential mechanism by which equine-assisted interventions produce positive outcomes, suggesting that the unique and supportive aspects of the equine context may be a therapeutic mechanism. In particular, the review suggests that the social aspects of the equine context offer a safe and nonjudgmental environment in which to develop interpersonal skills and secure supportive connections with other humans [35–37, 39, 41]. Indeed, a recent study that did not meet inclusion criteria due to its publication in 2018 found that incorporating veterans’ partners into an equine-assisted intervention offered unique benefits [64]. In addition, the horse-human bond was identified in 50% of the reviewed studies and may also be a fruitful target for theoretical development. Conceptual work elucidating the unique benefits of the horse-human bond [65] indicates that veterans with psychiatric conditions such as PTSD can more easily establish a connection with a horse compared to humans, thereby fulfilling emotional needs. Furthermore, horses are hypervigilant and sensitive to nonverbal cues; interacting with horses tends to encourage veterans to foster awareness of outward expressions of emotional dysregulation (e.g., aggressive movements) and translate that awareness to human interaction.

The above proposed mechanisms indicate that theoretical development for equine-assisted interventions among veterans may benefit from adopting conceptual frameworks that recognize the power of context in supporting desired outcomes [66, 67]. For example, such frameworks can be used to understand how the supportive and interactive qualities of the equine context may be capable of addressing the paradoxical relationship between psychiatric conditions and the supportive presence of others among veterans. On the one hand, the supportive presence of others is considered a buffer against PTSD [68], depression [69], and suicidal behavior [70]. On the other hand, the cognitive and emotional impairments that often accompany psychiatric conditions tend to limit the interpersonal skills veterans must employ to secure these critical connections with others [11, 71]. The safe and nonjudgmental nature of the equine context may offer an opportunity to interact with and secure support from other humans (i.e., participants and staff). Similarly, interacting with the horse offers an opportunity to experience an emotional connection and develop critical interpersonal skills in a nonthreatening context.

Among the reviewed studies, the aspect of theoretical development related to *who* benefits from intervention would benefit from a more comprehensive profile of veteran participants [63]. For example, of the six reviewed studies, only two [35, 37] included a standardized assessment of PTSD symptoms to understand their respective samples. Furthermore, neither of those studies offered a precise profile of the pattern of PTSD symptoms experienced by the participants, despite evidence [72] suggesting that veterans tend to experience a unique pattern of one or more of the following distinct symptom clusters: re-experiencing (e.g., emotional reactivity to trauma cues); avoidance (e.g., avoidance of thoughts); dysphoria (e.g., detachment); and hyperarousal (e.g., hypervigilance). Future research should include a detailed profile of veterans’ experience of the above symptom clusters, which may subsequently guide individualization of the intervention to target particular manifestations of symptom patterns. For example, a veteran experiencing the dysphoria symptom cluster may benefit from equine-assisted intervention that emphasizes facilitating a connection with the horse or other humans to address a sense of detachment. For veterans reporting symptoms consistent with the re-experiencing symptom cluster, intervention may emphasize using the horse’s sensitivity to outward expressions of emotional dysregulation to foster awareness of reactivity to trauma cues, a critical component of developing effective coping skills [73]. Similarly, none of the included studies sought to systematically understand the presence of comorbid conditions in their sample (e.g., depression and/or TBI). Given that PTSD often co-occurs with other diagnoses such as depression and TBI among veterans [2], future work should seek to understand how equine-assisted interventions can be individualized to address particular combinations of comorbid health conditions and whether responses to treatment vary according to particular combinations of comorbid conditions.

Current evidence for equine-assisted interventions for veterans would also be strengthened by a more comprehensive and thorough description of the intervention being tested. While all six studies *listed* the intervention components, these studies provided insufficient detail with respect to important aspects of describing an intervention, including the therapeutic goal of each individual component, whether the intervention was intended to be personalized or adapted to achieve individualized objectives, and to what extent intervention fidelity was achieved [74]. A more thorough description of the intervention would support the process of theoretical development by describing and testing an explicit link from one or more components of the intervention to therapeutic goals [63]. Furthermore, a more thorough description of the intervention and an evaluation of treatment fidelity can support successful implementation of the intervention by facilitating the identification of potential barriers to implementation and by providing clinicians with the detail required to employ a novel intervention in routine clinical practice [23, 74].

The implementation of equine-assisted interventions for veterans would also be supported by a greater understanding of both the factors contributing to attrition and the dose-response relationship. Only two included studies reported attrition rates, with one reporting a rate of 24% [35] and the other reporting a rate of 46% [41]. Furthermore, only one of these studies [41] reported factors *explaining* attrition, which included participants moving or becoming too busy to attend sessions. A more comprehensive understanding of the reasons for attrition would facilitate the execution of appropriate modifications to support the successful implementation of equine-assisted interventions for veterans. Finally, the paucity of studies, combined with the highly variable duration and length of included studies (see Table 2), precludes an accurate understanding of the most efficient dose-response relationship for equine-assisted interventions for veterans. An understanding of the optimal amount of treatment that tends to generate desired outcomes is a critical component of developing and evaluating interventions and must be achieved to ensure cost-effective, safe, and effective services for veterans [40, 63].

### Limitations

The search procedure employed in this study was sufficiently broad in scope to retrieve papers related to equine-assisted interventions among veterans, but we may have missed sources not indexed in databases targeted by our search or articles that were indexed after the final search in 2018. Indeed, we briefly addressed several articles [57–59, 64] that were published too recently to be included in this review. Because we restricted our search to studies published in the English language, we may have excluded pertinent studies written in other languages. Systematic mapping reviews do not typically include the formal assessment of the rigor of included studies, and as such, we did not conduct such an assessment. An assessment of the methodological quality of each individual study may be a worthy pursuit in future research. Finally, the representations of equine-assisted interventions among veterans included in this review were derived from peer-reviewed research studies and should not be considered a comprehensive reflection of the nature of equine-assisted interventions for veterans.

## Conclusions

Ideally, the approach to developing, evaluating, and implementing novel and complex equine-assisted interventions for the veteran population would be systematic and phased, beginning with a thorough understanding of the existing literature. However, while over 300 programs currently deliver equine-assisted services to veterans [20], little is known regarding the safe and effective delivery of such services. This systematic mapping review consequently sought to develop a comprehensive map of the body of evidence concerning equine-assisted interventions for veterans. Because various equine-assisted interventions that targeted psychosocial outcomes among veterans were found to be promising, they warrant continued scientific investment to help establish their efficacy. However, considerable gaps in knowledge or research approaches must also be addressed. Specifically, targeted outcomes should be expanded to include those consistent with polytraumatic injuries, including concurrent psychosocial (e.g., PTSD symptoms) and physical (e.g., pain) concerns. More nuanced perspectives on qualities of social functioning, self-referential outcomes (e.g., self-efficacy), and community reintegration are also needed. Future studies must also emphasize the theoretical development of equine-assisted interventions for veterans and thoroughly describe the participants, components of the intervention, factors contributing to attrition, and optimal dose-response relationships. Advancing knowledge of these phenomena will facilitate the identification of those interventions most worthy of widespread implementation on behalf of the veteran population.

## Data Availability

Not applicable.

## Abbreviations

AAIs: Animal-assisted interventions
BDI-II: Beck Depression Inventory II
BELT: Benefitting from Experiential Learning Together
CAPS: Clinician Administered PTSD Scale
CSES: Coping Self Efficacy Scale
CT-NOS: Certified trainer not otherwise specified
DERS: Difficulties in Emotion Regulation Scale
DET: Data extraction tool
DQ: Demographic Questionnaire
DSM-V: Diagnostic and Statistical Manual of Mental Disorders, Fifth Edition
EAAs: Equine-assisted activities
EAL: Equine-assisted learning
EATs: Equine-assisted therapies
EFMH: Equine facilitated mental health,
EFMHE: Equine facilitated mental health expert
HOLSTER: Horses Relieving Operational Stress through Experiential Relationships
HPOT: Hippotherapy
ICF: International Classification of Functioning, Disability, and Health
ICF-A/P: Activity/participation
ICF-BF: Body functions
MSSS: Modified Social Support Survey
NDI: Neck Disability Index
NOS: Not otherwise specified
OLBPQ: Oswestry Low Back Pain Questionnaire
OSI: Operational stress injury
PATH Intl: Professional Association of Therapeutic Horsemanship International
PCL-C: PTSD Checklist Civilian Version
PCL-M: PTSD Checklist Military Version
PT: Physical therapist
PTSD: Posttraumatic stress disorder
QOLI: Quality of Life Inventory
RSES: Response to Stressful Experiences Scale
SDS: Sheehan Disability Scale
SF-36v2: SF-36v2 Quality of Life Assessment
SW: Social worker
TBI: Traumatic brain injury
THR: Therapeutic horseback riding
TRI: Therapeutic riding instructor
WHODAS: World Health Organization Disability Assessment Schedule
